# Portal Vein Pulsatility Index as a Potential Risk of Venous Congestion Assessed by Magnetic Resonance Imaging: A Prospective Study on Healthy Volunteers

**DOI:** 10.3389/fphys.2022.811286

**Published:** 2022-04-29

**Authors:** Osama Abou-Arab, Christophe Beyls, Mouhamed Djahoum Moussa, Pierre Huette, Elodie Beaudelot, Mathieu Guilbart, Bruno De Broca, Thierry Yzet, Hervé Dupont, Roger Bouzerar, Yazine Mahjoub

**Affiliations:** ^1^ Anesthesia and Critical Care Department, Amiens Hospital University, Amiens, France; ^2^ CHU Lille, Pôle d’Anesthésie-Réanimation, Lille, France; ^3^ Department of Radiology, Amiens Picardy University Hospital, Amiens, France; ^4^ Department of Biophysics and image processing, Amiens Picardy University Hospital, Amiens, France

**Keywords:** fluid responsiveness, portal vein pulsatility, fluid challenge, venous congestion, magnetic resonance imaging

## Abstract

High values of the portal vein pulsatility index (PI) have been associated with adverse outcomes in perioperative or critically ill patients. However, data on dynamic changes of PI related to fluid infusion are scarce. We aimed to determine if dynamic changes in PI are associated with the fluid challenge (FC). To address this challenge, we conducted a prospective single-center study. The population study included healthy subjects. FC consisted in the administration of 500 ml of Ringer lactate infusion over 5 min. The portal blood flow and PI were assessed by magnetic resonance imaging. The responsiveness to FC was defined as an increase in the cardiac stroke volume of at least 10% as assessed by echocardiography. We included 24 healthy volunteers. A total of fourteen (58%) subjects were responders, and 10 (42%) were non-responders. In the responder group, FC induced a significant increase in portal blood flow from 881 (762–1,001) at the baseline to 1,010 (778–1,106) ml min^−1^ (*p* = 0.005), whilst PI remained stable (from 31 [25–41] to 35 (25–42) %; *p* = 0.12). In the non-responder group, portal blood flow remained stable after FC (from 1,042 to 1,034 ml min^−1^; *p* = 0.084), whereas PI significantly increased from 32 (22–40) to 48% *(25–85) after FC (*p* = 0.027). PI was negatively correlated to portal blood flow (Rho coefficient = −0.611; *p* = 0.002). To conclude, PI might be a sensitive marker of early congestion in healthy subjects that did not respond to FC. This finding requires further validation in clinical settings with a larger sample size.

## Introduction

The portal vein pulsatility index (PI) is a promising parameter to assess the hemodynamic status of the venous system. The PI is the ratio between minimal and maximal variation of the portal blood velocity over the cardiac cycle. In a physiological or healthy state, the pulsatility of the portal flow velocity is minimal (Gallix et al., 1997). Variations in velocity reflect variations in a pressure gradient. In a non-congestive venous system, changes in the venous pressure gradient over the cardiac cycle as a result of atrial contraction and relaxation are dampened due to venous compliance, and velocity remains relatively constant. On the other hand, in a non-compliant venous system, atrial pressure changes are directly transmitted upstream and result in venous velocity variations over the cardiac cycle (Hu et al., 2003) (Shih et al., 2006). There is no consensus on the normal range of PI. However, a value above 50% is usually considered to reflect abnormal venous congestion (Görg et al., 2002).

Numerous observational studies highlighted the association between high PI over 30% and adverse outcomes following cardiac surgery or in critically ill patients (Eljaiek et al., 2019) (Beaubien-Souligny et al., 2018) (Spiegel et al., 2020) (Benkreira et al., 2019). Nevertheless, it seems that PI measurement could provide a qualitative estimation of the central venous pressure. Indeed, central venous pressure is the standard approach and the most reported parameter to describe venous congestion. In a recent meta-analysis, high central venous pressure was significantly related to organ failure through venous congestion (Chen et al., 2020).

As the portal venous waveform is conditioned by backward transmission of right atrial intramural pressure, any variation in volemia might induce variations in PI. So far, no data are available on fluid infusion and PI variation. In addition, data on dynamic changes of PI related to acute fluid loading are scarce.

The portal vein is assessable using Doppler echography, but its low reproducibility and high variability make the assessment of changes in portal hemodynamics more difficult (Yzet et al., 2010a). In our institution, we have previously described the portal vein blood flow using magnetic resonance imaging (MRI), and we have found that PI is around 18 ± 6% in healthy subjects (Yzet et al., 2010b).

In order to precisely describe the portal flow and the PI, we decided to use MRI rather than Doppler ultrasound to avoid intra-observer variability and poor reproducibility.

The aim of our study was to describe dynamic PI changes induced by the fluid challenge (FC) using MRI.

## Material and Methods

### Ethics

This study was conducted according to the Declaration of Helsinki on clinical research on healthy volunteers. According to the French law on clinical research, the study was approved by the Institutional Review Board, and each participant provided written informed consent (CPP Sud Mediterranée III, France; reference PI 2018–843–0,006; chairperson: Lavabre-Bertrand on 6th June of 2018) (Toulouse et al., 2018). The study was registered on ClinicalTrial.gov (registration number: NCT03589261). The study was also approved by the French drug agency (Agence Nationale de Sécurité du Médicament et des Produits de Santé; reference 2018-A00729-46 on fourth of May 2018). We conducted a prospective single-center study at Amiens University Hospital. The present report was drafted in line with the STROBE statement for cohort studies (von Elm et al., 2007).

### Study Population

The study population consisted of healthy male volunteers, aged between 18 and 30 years. All subjects were fasting for 12 h before participation. Exclusion criteria were as follows: any pre-existing cardiac or liver disease, history of abdominal surgery, claustrophobia, and any contraindication to MRI.

### Assessment of Portal Blood Flow and Pulsatility Index by Magnetic Resonance Imaging (Figures 1, 2)

We used MRI to assess portal blood flow and PI. MRI was performed using a 3T scanner (Achieva dStream, Philips Healthcare, Best, Netherlands) with a 16-channel-phased array coil (dS torso). Coronal and axial breath-hold-balanced gradient-echo images were acquired in order to localize the appropriate orientation of the oblique slice required for portal blood flow measurements (ESM Figures 1A, B). Velocity images were acquired using gradient-echo phase-contrast pulse sequences (PCMRI) with inspiratory breath-hold and retrospective cardiac gating (peripheral plethysmograph). PCMRI was acquired in a plane orthogonal to the portal vein axis using an encoding velocity Venc = 40 cm/s based on the previous work (Yzet et al., 2010a) (Yzet et al., 2010b). This encoding velocity is a scale amplitude, chosen before performing the MR sequence so as to encompass the highest blood velocity likely to be encountered inside the vessel studied. Each series of reconstructed data consisted of magnitude images associated with the corresponding phase images, in which the pixel intensity is linearly related to the local tissue velocity (in-plane resolution: 0.9 mm^2^× 0.9 mm^2^). The flow rates were calculated from 16 velocity images spanning the cardiac cycle.

**FIGURE 1:**
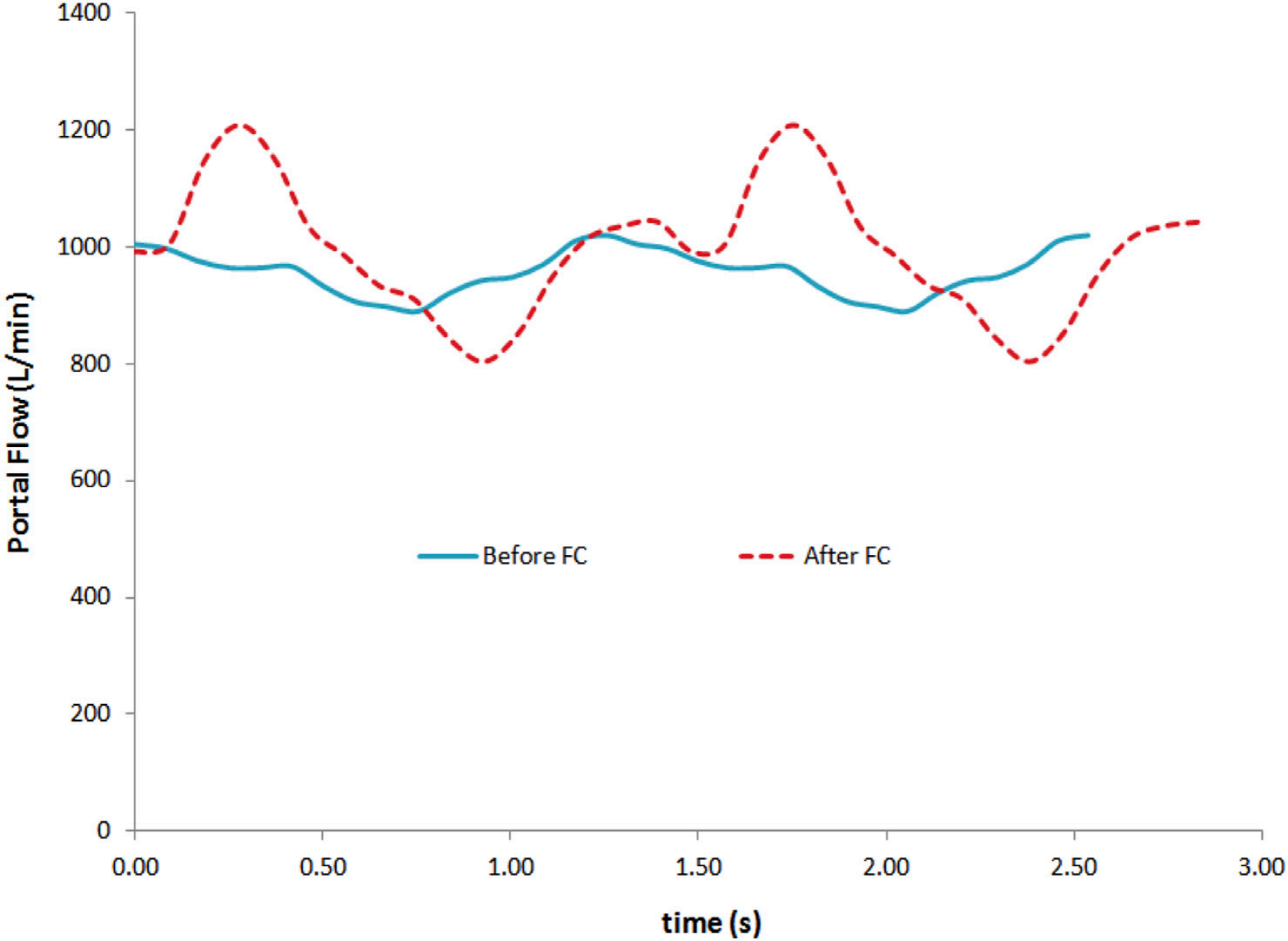
Portal flow rate from a subject before (continuous) and after (dotted line) the fluid challenge (FC) during two cardiac cycles in a non-responder subject. The curve signal evolved from low to high pulsatility.

**FIGURE 2:**
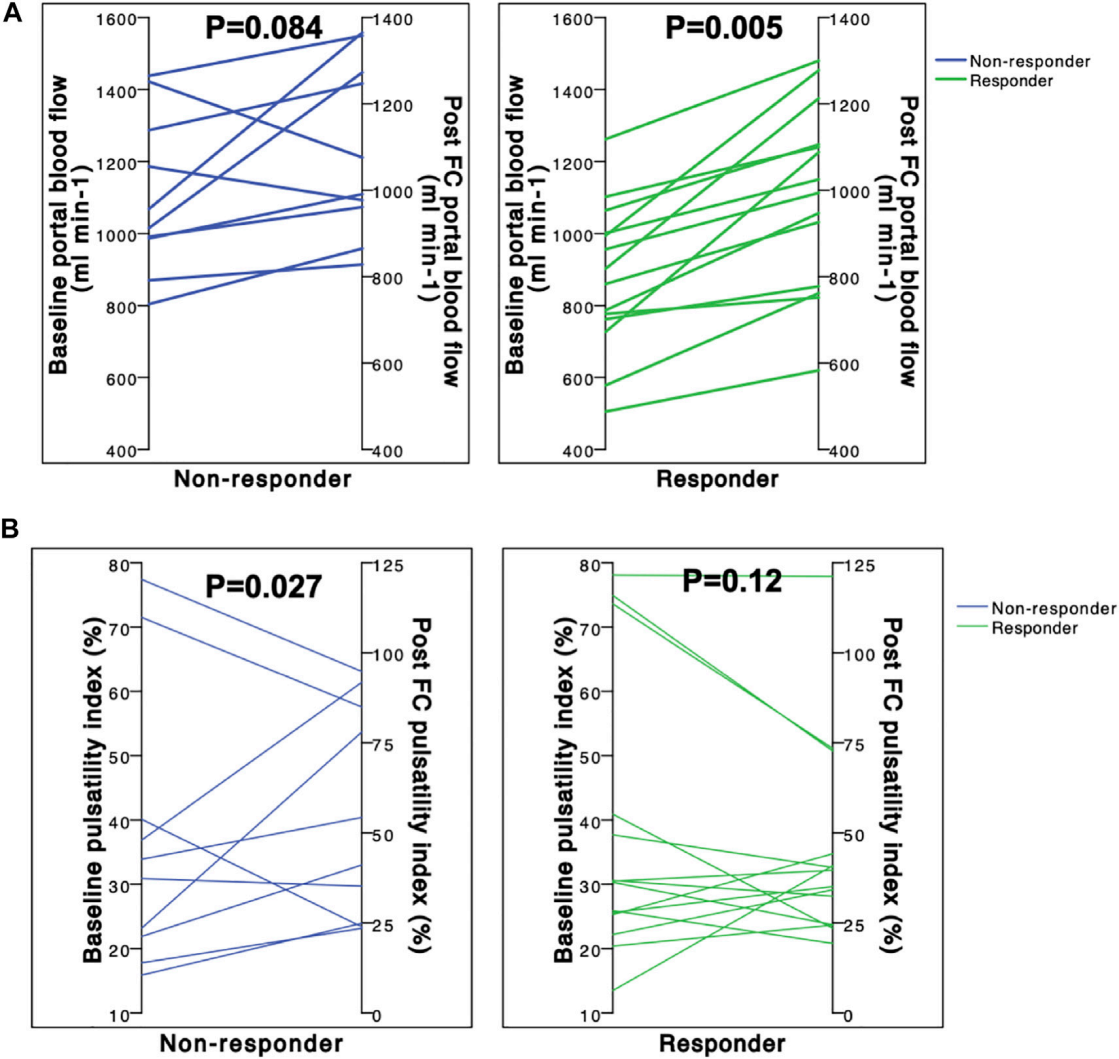
Box plots showing changes in the portal blood flow **(A)** and portal vein pulsatility index **(B)** at baseline and after fluid challenge (FC). Fluid responsiveness was defined by a greater than 10% increase in stroke volume. Portal blood flow (ml.min−1) was measured by portal vein MRI. The pulsatility index (%) was calculated as 100^∗^(maximum portal velocity-minimum portal velocity)/maximum portal velocity. Baseline/post-FC comparisons were performed using a Wilcoxon rank sum test. P: *p*-value for the statistical test.

**FIGURE 3:**
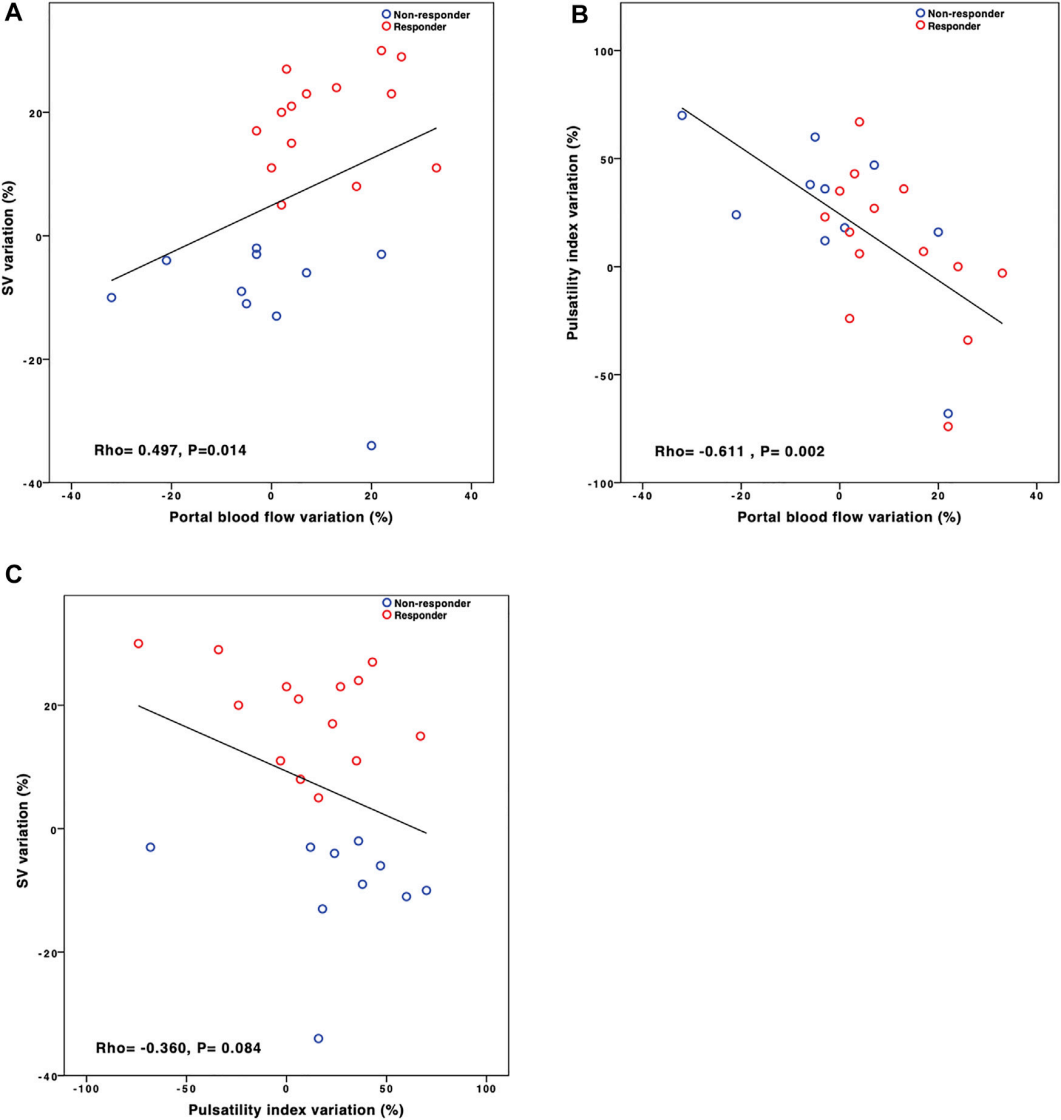
Relationships between the stroke volume (SV), pulsatility index, and portal blood flow variations induced by the fluid challenge (FC). Fluid responsiveness was defined by a greater than 10% increase in SV. Variation was calculated for each variable as 100^∗^(post-FC value-baseline value)/post-FC value. Red plots: responder. Blue plot: non-responder. Correlations were tested using Spearman’s correlation test.

MRI data were initially transferred in a DICOM format to a dedicated workstation. Flow quantification and analysis were performed using freely available software (Segment, Medviso, Lund, Sweden) with deformable contours in order to match the variation of the vessel section throughout the cardiac cycle (Figure 1) [3]. During the post-processing step, this tool allows semi-automatic extraction of temporal flow curves Q (t) from the velocity pixels inside the region of interest (ROI). Hemodynamic parameters, such as maximum and mean velocities, vessel cross-sectional area, and flow rate at each phase of the cardiac cycle, can therefore be extracted. The flow rate is defined as the product of the vessel cross-sectional area and the mean velocity inside the lumen.

Two hemodynamic indices were derived from these measured data:

- Portal blood flow (ml min^−1^) was defined as the product of the vessel cross-sectional area and the mean velocity inside the lumen.
- PI (%) was calculated from the maximum, minimum, and mean blood flow velocity and defined as the ratio (maximum portal blood flow velocity-minimum portal blood flow velocity)/mean portal blood flow velocity (Figure 1 and ESM video). (Boote, 2003).

### Echocardiography Measurement and Fluid Challenge Procedure

Transthoracic echocardiography (CX50 Ultrasound System, Philips Medical System, Suresnes, France) was performed by a single experienced cardiologist and using standard views in accordance with current guidelines immediately before and 10 min after the fluid challenge (FC) (Evangelista et al., 2008). A measure of 500 ml of lactated Ringer solution was administered. Measurements were performed offline by the same cardiologist blinded to MRI data.

Left ventricular (LV) ejection fraction was calculated using the modified Simpson’s method from apical four and two-chamber views. From pulsed-wave Doppler measurements of transmitral velocities, the peak E velocity (E wave), peak A velocity (A wave), ratio between the peak E and A velocities (E/A ratio), and deceleration time (E deceleration) were measured. From the Doppler tissue imaging, lateral e’ and lateral E/e’ ratios were measured.

RV fractional area change (%) was measured from end systolic and end diastolic changes in the RV area in a four-chamber view as follows: 100 x (end diastolic area-end systolic area)/end diastolic area.

Using pulsed-wave Doppler, we measured sus hepatic S wave, sus hepatic D wave, and the sus hepatic ratio (S/D).

Inferior vena cava (IVC) collapsibility (%) was measured as follows: IVC collapsibility (%) = 100 × (expiratory IVC–inspiratory IVC)/expiratory IVC.

From the 5-chamber view, the velocity of the flow in the left ventricular outflow tract was acquired using pulsed-wave Doppler. The velocity–time integral was measured and averaged over three consecutive cardiac cycles (Fischer et al., 2014). In a long axis parasternal view, the chamber area was calculated from the left ventricular outflow tract diameter. The intra-observer variability of the velocity–time integral was 3 ± 1%.

### Definition of Fluid Responsiveness

The subjects were defined as fluid responders (R group) if their SV increase by at least 10% after FC (Cecconi et al., 2014) (Guinot et al., 2014). The others are defined as non-responders (NR group). The assessment of responsiveness was approximately performed 15–20 min after FC, once the MRI process was performed.

We also compared PI variation using other threshold values (15 and 20% of SV increase) to differentiate between responders and non-responders. (See Supplementary File S1).

### Statistical Analysis

Data are expressed as median (interquartile range) or numbers (percentage), as appropriate. Variables were compared using Wilcoxon–Mann–Whitney, Chi-square, or Wilcoxon rank sum tests, as appropriate. SV, portal blood flow, and PI variations were calculated as follows: (%) = 100*(post FC value-baseline value)/baseline value for each variable. Correlations between SV, PI, and portal blood flow were assessed using the non-parametric Spearman correlation test. All statistical analyses were performed using IBM SPSS software (SPSS, version 24, IBM, New York, NY). The limit of statistical significance was *p* < 0.05.

## Results

### Demographic Data (Table 1)

Demographic data and clinical characteristics are presented in Table 1. A total of twenty-four healthy male volunteers were included from September 2018 to January 2019. Among those, 14 patients (58%) were responders (R group) after 500 ml of FC. Median age and BMI were similar between groups.

**TABLE 1:** Demographics data. Responders were defined by an increase in stroke volume of 10% after a fluid challenge of 500 ml. BMI: body mass index.

Variable	Non-responder (*n* = 10)	Responder (*n* = 14)	*p*-value
Age; years	27 (25–28)	28 (26–28)	0.259
BMI; kg m^−2^	23.6 (22.1–24.3)	23.4 (21.5–24.6)	0.625
Body surface area; m^2^	2.21 (2.05–2.24)	1.99 (1.91–2.08)	0.052

**TABLE 2:** Clinical data and left ventricular (LV) and right ventricular (RV) echocardiographic parameters in non-responders and responders after 500 ml of fluid challenge (FC). SAP: systolic arterial pressure; DAP: diastolic arterial pressure; MAP: mean arterial pressure; HR: heart rate; IVC: inferior vena cava; TAPSE: tricuspid annular plane systolic excursion; VTI: velocity–time integral.

Variable	Non-responder (n = 10)	Responder (n = 14)	*p*-value
**Clinical data**
SAP, mmHg	—	—	—
Baseline	128 (125–137)	118 (115–127)	0.192
After FC	121 (119–126)	117 (109–126)	0.259
DAP, mmHg	—	—	—
Baseline	71 (62–84)	64 (62–68)	0.259
After FC	64 (60–75)	66 (61–79)	0.508
MAP, mmHg	—	—	—
Baseline	83 (80–87)	76 (73–81)	**0.028**
After FC	77 (72–85)	77 (72–85)	0.886
HR, bpm	—	—	—
Baseline	67 (65–75)	63 (60–73)	0.508
After FC	62 (60–67)	63 (56–68)	0.796
**LV echocardiography**			
VTI aortic, cm s^−1^	—	—	—
Baseline	23.5 (22.0–24.0)	19.0 (17.0–20.0)	**0.001**
After FC	21.0 (18.0–23.0)	24.5 (20.0–25.0)	0.108
Stroke volume, ml	—	—	—
Baseline	76 (71–91)	65 (53–79)	**0.048**
After FC	70 (62–97)	78 (62–97)	0.508
E-wave, cm s^−1^	—	—	—
Baseline	96 (80–101)	76 (73–84)	**0.019**
After FC	74 (62–93)	88 (80–104)	0.508
E/A ratio	—	—	—
Baseline	1.9 (1.7–2.6)	1.8 (1.5–1.9)	0.312
After FC	2.2 (1.4–2.4)	2.1 (1.7–2.6)	0.977
E deceleration time, ms	197 (171–234)	236 (175–333)	0.192
Before/after FC	192 (157–283)	234 (198–274)	0.508
Lateral E/e’	—	—	—
Baseline	5.5 (4.0–6.0)	4.0 (4.0–4.0)	0.186
After FC	4.0 (4.0–4.0)	5.0 (4.0–5.0)	0.056
**RV echocardiography**			
RV fractional area change, %	—	—	—
Baseline	39 (35–43)	48 (40–52)	0.212
After FC	42 (35–45)	43 (36–47)	0.539
TAPSE, mm	—	—	—
Baseline	24 (21–29)	22 (20–27)	**0.043**
After FC	26 (23–29)	25 (23–27)	0.508
S-wave, cm s^−1^	—	—	—
Baseline	15 (14–17)	14 (11–14)	0.046
After FC	14 (13–16)	14 (13–16)	0.927
IVC min diameter, mm	—	—	—
Baseline	12 (10–14)	11 (10–14)	0.841
After FC	12 (11–14)	16 [11–16)^a^	0.212
IVC max diameter, mm	—	—	—
Baseline	20 (17–21)	18 (16–22)	0.585
After FC	21 (17–23)	19 (17–22)	0.472
IVC collapsibility, %	—	—	—
Baseline	38 (18–48)	32 (23–50)	0.709
After FC	36 (29–45)	21 (11–36)	0.074
Sus hepatic S/D ratio	—	—	—
Baseline	1.33 (1.07–1.55)	1.16 (0.96–1.39)	0.600
After FC	1.35 (1.26–1.67)	1.25 (1.05–1.58)	0.285

### Clinical and Hemodynamic Data Before and After Fluid Challenge (Table 2)

HR, SAP, and DAP were similar between groups before and after FC. MAP was significantly lower in the responder group than that in the non-responder group [respectively, 76 (73–81) vs. 83 (80–87) mmHg; *p* = 0.028]. After FC, MAP was similar between the two groups (Table 2).

SV was lower in the R group than in the NR group [respectively, 65 (53–79) vs. 76 (71–91) ml; *p* = 0.048] and was similar after FC in both groups (*p* = 0.508).

RV systolic functions including TAPSE, S-wave, and RV FAC were similar between the two groups before and after FC.

The IVC collapsibility was similar between the two groups before FC [respectively, in the NR and R groups, 38 (18–48) vs. 36 (29–45) %; *p* = 0.709]. After FC, IVC collapsibility remained unchanged for the R group from 32 (23–50) to 21% (11–36) with *p* = 0.079 and the NR group [38 (18–48) vs. 36 (29–45) %; *p* = 0.635].

### Magnetic Resonance Imaging Portal Hemodynamics Before and After Fluid Challenge (Table 3 and Figure 2)

At the baseline, portal blood flow was lower in the R group than that in the NR group [881 (762–1,001) vs. 1,042 (986–1,287) ml min^−1^; *p* = 0.022). Portal blood flow significantly increased in the R group (*p* = 0.005) after FC but not in the NR group (*p* = 0.084).

PI was similar between the two groups before FC. After FC, PI significantly increased in the NR group [from 32 (22–40) to 48 (25–85) %; *p* = 0.027], whereas it remains similar in the R group [from 31 (25–41) to 35 (25–42) %; *p* = 0.12] (Figure 2).

The portal velocity and portal vein section area remained similar in the two groups before and after FC.

### Relationships Between Portal Blood Flow, Pulsatility Index, and Stroke Volume Variations (Figure 2)

SV variation was positively correlated with portal blood flow variation (Rho = 0.494; *p* = 0.014) and not negatively correlated with PI variation (Rho = −0.360; *p* = 0.084) (Figure 3).

PI variation was strongly negatively correlated with portal blood flow variation (Rho = −0.611; *p* = 0.002).

## Discussion

Here, we report several findings. Both portal blood flow and PI changes were induced by FC, but these changes depended on the fluid responsiveness of the subject. In responders, the portal blood flow increased with SV, whereas PI remained unchanged. In the NR group, PI increased after FC, and the portal blood flow remained unchanged.

### Dynamic Changes in the Pulsatility Index

Based on the results of our study, it seems that PI is influenced by the volume status with an increase in pulsatility related to fluid overload. To the best of our knowledge, only two previous reports have emphasized the relationship between PI and volume status (Singh et al., 2019) (Bouabdallaoui et al., 2020). In these reports, treatment with loop diuretics at hospital admission in patients with high PI (above 50%) and acute heart failure allowed clinical improvement associated with the PI decrease. In our report, PI significantly increased from 32 to 48% in the NR group, confirming the relationship between venous congestion and PI.

Others markers such as inferior vena cava diameter can also reflect volemic status. A low or high inferior vena cava diameter can reflect hypovolemia or venous congestion (Zengin et al., 2013) (Guinot et al., 2015). Combined with the Doppler waveform of veins (femoral, renal, or portal veins), ultrasound scores were proposed to quantify venous congestion (Beaubien-Souligny et al., 2020). Regardless of the chosen vein, the concept of the waveform shift according to volemia changes seems promising. A recent study on healthy subjects showed an increase in basilica vein pulsatility induced by a passive leg raising (Ermini et al., 2021). The observation confirms that the ability of vein pulsatility shifts to pressure/volume variation. Unfortunately, we did not assess other markers as renal of hepatic flow patterns. In addition, IVC collapsibility was not different between groups before and after FC (Table 3) which highlights the fact that non-responders did not reach an overt status of venous congestion. We may suggest that there is a continuum from a preload dependency state to a venous congestion state. Moreover, a FC of only 500 ml was probably not enough to observe typical signs of venous congestion as changes in IVC.

**TABLE 3:** Comparisons of MRI portal hemodynamic between non-responders and responders after 500 ml of fluid challenge. The bold value corresponds to p-value < 0.05.

Variable	Non-responder (*n* = 10)	Responder (*n* = 14)	*p*-value
**Portal flow, ml min^−1^**	—	—	—
Baseline	1,042 (986–1,287)	881 (762–1,001)	**0.022**
After FC	1,034 (961–1,273)	1,010 (778–1,106)^a^	0.371
**Portal velocity, cm s^−1^**	—	—	—
Baseline	24.0 (20.8–29.9)	20.8 (18.7–26.6)	0.122
After FC	26.4 (22.1–30.1)	21.4 (19.6–26.9)	0.212
**Pulsatility index, %**	—	—	—
Baseline	32 (22–40)	31 (25–41)	0.931
After FC	48 (25–85)^a^	35 (25–42)	0.312
**Portal vein cross-sectional area, cm^2^**	—	—	—
Baseline	1.7 (1.6–1.9)	1.5 (1.4–1.7)	0.064
After FC	1.8 (1.5–2.1)	1.6 (1.5–1.7)	0.437

Data are expressed as median (interquartile range). SV: stroke volume. Pulsatility index was calculated as 100*(maximum portal velocity-minimum portal velocity)/maximum portal velocity. a: p-value < 0.05 for baseline/post-FC, comparisons using the Wilcoxon rank sum test. The bold value corresponds to p-value < 0.05.

### Portal Blood Flow and Venous Return

Our experimental schema supposes that portal blood flow would reflect venous return. In an experimental model of anesthetized dogs, the authors showed that the portal blood flow counts for 20% of the cardiac output and reported a good correlation (r = 0.88) between these flows (Smith et al., 1986). However, in spite of a greater volemia after FC, the NR group did not increase either its portal blood flow or its cardiac output. Hence, we may hypothetize that the stressed volume was not reached enough to allow an increase in cardiac output in this group. The portal blood flow will increase only if the FC counts for a stressed volume as reflected in the R group.

Given these observations, the estimation of preload dependency by the portal blood flow seems inappropriate. Certainly, the portal blood flow plays a role in venous return but monitoring of portal blood flow solely seems inadequate and inaccurate to assess venous return.

On the contrary, high portal vein pulsatility appears as a maker to stop any infusion of extra fluid.

### Use of Magnetic Resonance Imaging to Evaluate Portal Blood Flow and Pulsatility Index

The second main point of our study was to assess the dynamic changes in portal vein induced by therapies such as FC. Indeed, most studies we mentioned reported PI as a static marker of prognosis. Small reports have shown that inhaled agents by improving right ventricular function and possible venous congestion can reduce portal pulsatility (Tremblay et al., 2017) (Couture et al., 2019).

Given the growing and robust data on venous congestion and portal vein pulsatility, the further perspective is to assess the reversibility of pulsatility at the bedside. MRI use allowed the before/after FC comparison. With phase-contrast imaging, the MRI signal is the reference method to visualize and quantify velocity (Wymer et al., 2020). Intra-individual variability reported in MRI measurements of the portal vein flow is 7% (Lycklama à Nijeholt et al., 1997). Hence, the before/after variation of FC can be used according to the effect of FC given the higher variation of the portal blood flow and PI. The accuracy Doppler echography measurement of the portal vein depends on various parameters that must be considered. First, the variation in the angle between the beam and the vessel increases the risk of error measurement. Second, the blood velocity might be influenced by the vessel deformation following FC. Hence, the area of the vessel might be different. Third, the formula measuring areas considers vessels as having a circular shape which may not necessarily reflect the real shape, especially after FC.

Given these limits, we preferred MRI use to Doppler echography to validate the concept of our study. However, in clinical settings, investigations at the bedside will require Doppler echography.

### Limits

Our study admits several limitations. First, we included only male subjects and not female subjects as a restriction from the French national drug agency (ANSM) to avoid misdiagnosis of pregnancy. However, we voluntarily restricted the age of the included volunteers between 20 and 30 to obtain the most homogenous population study given the limited sample size. The other advantage of that restriction was to avoid physiological changes related to age.

Echocardiography might represent a limit in SV measurement. However, all SV measurements were performed by the same experienced cardiologist. In addition, the intra-observer variability of velocity–time integral was 3 ± 1%, which was lower than the threshold of SV used to assess fluid responsiveness. Last, a recent meta-analysis confirmed that assessing changes in SV was accurate and reliable for spontaneous breathing subjects (Chaves et al., 2018). We did not monitor the cardiac output and SV in a continuous way with non-invasive devices because, to our knowledge, no one was MRI-safe.

Our percentage of fluid responsiveness of 58% might be surprising in spite of the narrow range of age and the standardized protocol study. The variability in fluid responsiveness for healthy subjects reported in the literature seems to be quite high (from 45 to 90%) even if the method used to assess SV differed between studies (Godfrey et al., 2014) (Kumar et al., 2004) (Miller et al., 2016). In the Godfrey *et al* study, using the same method of SV measurement and a similar healthy subject population, the authors reported a percentage of fluid responsiveness of 45%. (Godfrey et al., 2014). So, these previous findings as well as our study support the fact that a healthy subject is not always a responder to fluid challenge.

The percentage of fluid responsiveness might depend on the rate of Ringer lactate infusion given that MRI measurements required an approximate time of 15–20 min. Nevertheless, in a dedicated study of volume kinetics in healthy subjects, Yi et al. showed that plasma dilution was efficient starting from five to more than 60 min after FC (Yi et al., 2019).

Last, PI changes could be induced by a modification of vein portal configuration after FC. In this way, we assessed the cross-sectional area, which remained similar in both groups after FC (Table 3).

In spite of these limitations, we believe that the main strength of the study is the MRI use which offered a good reproducibility of measurements before/after FC, at the precise same section of the portal vein as the subject remained in the same position during the whole procedure.

## Conclusion

For healthy subjects, when a fluid loading was not followed by the stroke volume increase, PI increases. Hence, a high portal pulsatility index seems to be associated with a potential risk of venous congestion. Further investigations are required to confirm the potential role of the portal pulsatility index at the bedside to assess the volume status of critically ill patients.

## Data Availability

The raw data supporting the conclusion of this article will be made available by the authors, without undue reservation.

## Abbreviations

FC, fluid challenge; MRI, magnetic resonance imaging; NR, non-responder; PI, pulsatility index; R, responder; SV, stroke volume.

## References

[B1] Beaubien‐Souligny W. Benkreira A. Robillard P. Bouabdallaoui N. Chassé M. Desjardins G. (2018). Alterations in Portal Vein Flow and Intrarenal Venous Flow Are Associated with Acute Kidney Injury after Cardiac Surgery: A Prospective Observational Cohort Study. Jaha 7, e009961. 10.1161/JAHA.118.009961 30371304 PMC6404886

[B2] Beaubien-Souligny W. Rola P. Haycock K. Bouchard J. Lamarche Y. Spiegel R. (2020). Quantifying Systemic Congestion with Point-Of-Care Ultrasound: Development of the Venous Excess Ultrasound Grading System. Ultrasound J. 12. 10.1186/s13089-020-00163-w PMC7142196 32270297

[B3] Benkreira A. Beaubien-Souligny W. Mailhot T. Bouabdallaoui N. Robillard P. Desjardins G. (2019). Portal Hypertension Is Associated with Congestive Encephalopathy and Delirium after Cardiac Surgery. Can. J. Cardiol. 35, 1134–1141. 10.1016/j.cjca.2019.04.006 31395469

[B4] Boote E. J. (2003). AAPM/RSNA Physics Tutorial for Residents: Topics in US. RadioGraphics 23, 1315–1327. 10.1148/rg.235035080 12975518

[B5] Bouabdallaoui N. Beaubien‐Souligny W. Denault A. Y. Rouleau J. L. (2020). Impacts of Right Ventricular Function and Venous Congestion on Renal Response during Depletion in Acute Heart Failure. ESC Heart Fail. 7, 1723–1734. 10.1002/ehf2.12732 32400036 PMC7373894

[B6] Cecconi M. De Backer D. Antonelli M. Beale R. Bakker J. Hofer C. (2014). Consensus on Circulatory Shock and Hemodynamic Monitoring. Task Force of the European Society of Intensive Care Medicine. Intensive Care Med. 40, 1795–1815. 10.1007/s00134-014-3525-z 25392034 PMC4239778

[B7] Chaves R. C. d. F. Corrêa T. D. Neto A. S. Bravim B. d. A. Cordioli R. L. Moreira F. T. (2018). Assessment of Fluid Responsiveness in Spontaneously Breathing Patients: a Systematic Review of Literature. Ann. Intensive Care 8, 21. 10.1186/s13613-018-0365-y 29427013 PMC5807252

[B8] Chen C.-Y. Zhou Y. Wang P. Qi E.-Y. Gu W.-J. (2020). Elevated central Venous Pressure Is Associated with Increased Mortality and Acute Kidney Injury in Critically Ill Patients: a Meta-Analysis. Crit. Care 24, 80. 10.1186/s13054-020-2770-5 32138764 PMC7059303

[B9] Couture E. J. Tremblay J.-A. Elmi-Sarabi M. Lamarche Y. Denault A. Y. (2019). Noninvasive Administration of Inhaled Epoprostenol and Inhaled Milrinone in Extubated, Spontaneously Breathing Patients with Right Ventricular Failure and Portal Hypertension: A Report of 2 Cases. A. A. Pract. 12, 208–211. 10.1213/XAA.0000000000000886 30216198

[B10] Eljaiek R. Cavayas Y. A. Rodrigue E. Desjardins G. Lamarche Y. Toupin F. (2019). High Postoperative portal Venous Flow Pulsatility Indicates Right Ventricular Dysfunction and Predicts Complications in Cardiac Surgery Patients. Br. J. Anaesth. 122, 206–214. 10.1016/j.bja.2018.09.028 30686306

[B11] Ermini L. Chiarello N. E. De Benedictis C. Ferraresi C. Roatta S. (2021). Venous Pulse Wave Velocity Variation in Response to a Simulated Fluid challenge in Healthy Subjects. Biomed. Signal Process. Control. 63, 102177. 10.1016/j.bspc.2020.102177

[B12] Evangelista A. Flachskampf F. Lancellotti P. Badano L. Aguilar R. Monaghan M. (2008). European Association of Echocardiography Recommendations for Standardization of Performance, Digital Storage and Reporting of Echocardiographic Studies. Eur. J. Echocardiography 9, 438–448. 10.1093/ejechocard/jen174 18579482

[B13] Fischer M.-O. Balaire X. Le Mauff de Kergal C. Boisselier C. Gérard J.-L. Hanouz J.-L. (2014). The Diagnostic Accuracy of Estimated Continuous Cardiac Output Compared with Transthoracic Echocardiography. Can. J. Anesth/j Can. Anesth. 61, 19–26. 10.1007/s12630-013-0055-z 24155127

[B14] Gallix B. P. Taourel P. Dauzat M. Bruel J. M. Lafortune M. (1997). Flow Pulsatility in the portal Venous System: a Study of Doppler Sonography in Healthy Adults. Am. J. Roentgenology 169, 141–144. 10.2214/ajr.169.1.9207514 9207514

[B15] Godfrey G. E. P. Dubrey S. W. Handy J. M. (2014). A Prospective Observational Study of Stroke Volume Responsiveness to a Passive Leg Raise Manoeuvre in Healthy Non-starved Volunteers as Assessed by Transthoracic Echocardiography. Anaesthesia 69, 306–313. 10.1111/anae.12560 24641636

[B16] Görg C. Riera-Knorrenschild J. Dietrich J. (2002). Colour Doppler Ultrasound Flow Patterns in the portal Venous System. Bjr 75, 919–929. 10.1259/bjr.75.899.750919 12466260

[B17] Guinot P.-G. Urbina B. de Broca B. Bernard E. Dupont H. Lorne E. (2014). Predictability of the Respiratory Variation of Stroke Volume Varies According to the Definition of Fluid Responsiveness. Br. J. Anaesth. 112, 580–581. 10.1093/bja/aeu031 24535513

[B18] Guinot P. G. Arab O. A. Longrois D. Dupont H. (2015). Right Ventricular Systolic Dysfunction and Vena Cava Dilatation Precede Alteration of Renal Function in Adult Patients Undergoing Cardiac Surgery. Eur. J. Anaesthesiology 32, 535–542. 10.1097/EJA.0000000000000149 25192267

[B19] Hu J.-T. Yang S.-S. Lai Y.-C. Shih C.-Y. Chang C.-W. (2003). Percentage of Peak-To-Peak Pulsatility of portal Blood Flow Can Predict Right-Sided Congestive Heart Failure. Wjg 9, 1828–1831. 10.3748/wjg.v9.i8.1828 12918130 PMC4611553

[B20] Kumar A. Anel R. Bunnell E. Habet K. Zanotti S. Marshall S. (2004). Pulmonary Artery Occlusion Pressure and central Venous Pressure Fail to Predict Ventricular Filling Volume, Cardiac Performance, or the Response to Volume Infusion in normal Subjects. Crit. Care Med. 32, 691–699. 10.1097/01.ccm.0000114996.68110.c9 15090949

[B21] Lycklama à Nijeholt G. J. Burggraaf K. Wasser M. N. J. M. Schultze Kool L. J. Schoemaker R. C. Cohen A. F. (1997). Variability of Splanchnic Blood Flow Measurements Using MR Velocity Mapping under Fasting and post-prandial Conditions - Comparison with echo-Doppler. J. Hepatol. 26, 298–304. 10.1016/S0168-8278(97)80045-1 9059950

[B22] Miller J. Ho C.-X. Tang J. Thompson R. Goldberg J. Amer A. (2016). Assessing Fluid Responsiveness in Spontaneously Breathing Patients. Acad. Emerg. Med. 23, 186–190. 10.1111/acem.12864 26764894

[B23] Shih C.-Y. Yang S.-S. Hu J.-T. Lin C.-L. Lai Y.-C. Chang C.-W. (2006). Portal Vein Pulsatility index Is a More Important Indicator Than Congestion index in the Clinical Evaluation of Right Heart Function. Wjg 12, 768–771. 10.3748/wjg.v12.i5.768 16521192 PMC4066129

[B24] Singh G. Rachoin J.-S. Chien C. Patel S. (20192019). The Use of Portal Vein Pulsatility to Differentiate Hypervolemic and Hypovolemic Hyponatremia. Case Rep. Crit. Care 2019, 1–4. 10.1155/2019/9591823 PMC6662413 31380122

[B25] Smith H.-J. Grøttum P. Simonsen S. (1986). Ultrasonic Assessment of Abdominal Venous Return. Acta Radiologica. Diagn. 27, 23–27. 10.1177/028418518602700105 3515855

[B26] Spiegel R. Teeter W. Sullivan S. Tupchong K. Mohammed N. Sutherland M. (2020). The Use of Venous Doppler to Predict Adverse Kidney Events in a General ICU Cohort. Crit. Care 24. 10.1186/s13054-020-03330-6 PMC7574322 33076961

[B27] Toulouse E. Masseguin C. Lafont B. McGurk G. Harbonn A. A Roberts J. J. (2018). French Legal Approach to Clinical Research. Anaesth. Crit. Care Pain Med. 37, 607–614. 10.1016/j.accpm.2018.10.013 30580775

[B28] Tremblay J.-A. Beaubien-Souligny W. Elmi-Sarabi M. Desjardins G. Denault A. Y. (2017). Point-of-Care Ultrasonography to Assess Portal Vein Pulsatility and the Effect of Inhaled Milrinone and Epoprostenol in Severe Right Ventricular Failure. A A Case Rep. 9, 219–223. 10.1213/XAA.0000000000000572 28604468

[B29] von Elm E. Altman D. G. Egger M. Pocock S. J. Gøtzsche P. C. Vandenbroucke J. P. (2007). The Strengthening the Reporting of Observational Studies in Epidemiology (STROBE) Statement: Guidelines for Reporting Observational Studies. Ann. Intern. Med. 147, 573–577. 10.7326/0003-4819-147-8-200710160-00010 17938396

[B30] Wymer D. T. Patel K. P. Burke W. F. Bhatia V. K. (2020). Phase-Contrast MRI: Physics, Techniques, and Clinical Applications. RadioGraphics 40, 122–140. 10.1148/rg.2020190039 31917664

[B31] Yi J.-M. Bang J.-Y. Choi B. Cho C. Lee Y.-H. Lee E.-K. (2019). Population-based Volume Kinetics of Crystalloids and Colloids in Healthy Volunteers. Sci. Rep. 9, 18638. 10.1038/s41598-019-55171-1 31819122 PMC6901468

[B32] Yzet T. Bouzerar R. Allart J.-D. Demuynck F. Legallais C. Robert B. (2010a). Hepatic Vascular Flow Measurements by Phase Contrast MRI and Doppler Echography: a Comparative and Reproducibility Study. J. Magn. Reson. Imaging 31, 579–588. 10.1002/jmri.22079 20187200

[B33] Yzet T. Bouzerar R. Baledent O. Renard C. Lumbala D. M. Nguyen-Khac E. (2010b). Dynamic Measurements of Total Hepatic Blood Flow with Phase Contrast MRI. Eur. J. Radiol. 73, 119–124. 10.1016/j.ejrad.2008.09.032 19008062

[B34] Zengin S. Al B. Genc S. Yildirim C. Ercan S. Dogan M. (2013). Role of Inferior Vena Cava and Right Ventricular Diameter in Assessment of Volume Status: a Comparative Study. Am. J. Emerg. Med. 31, 763–767. 10.1016/j.ajem.2012.10.013 23602752

