# Supplementary figures and images for: Portal Vein Pulsatility Index as a Potential Risk of Venous Congestion Assessed by Magnetic Resonance Imaging: A Prospective Study on Healthy Volunteers
Source: Front Physiol. 2022 Apr 29;13:811286. doi: 10.3389/fphys.2022.811286 (PMC9101294; doi:10.3389/fphys.2022.811286)

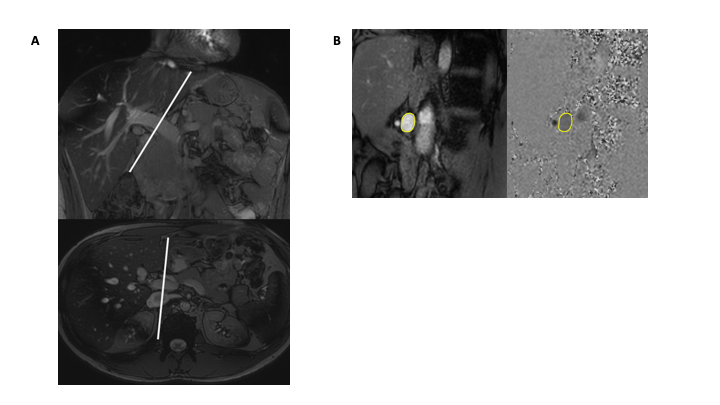

Supplement: Supplementary file 1 [file Image1.TIFF]

## Slide 1
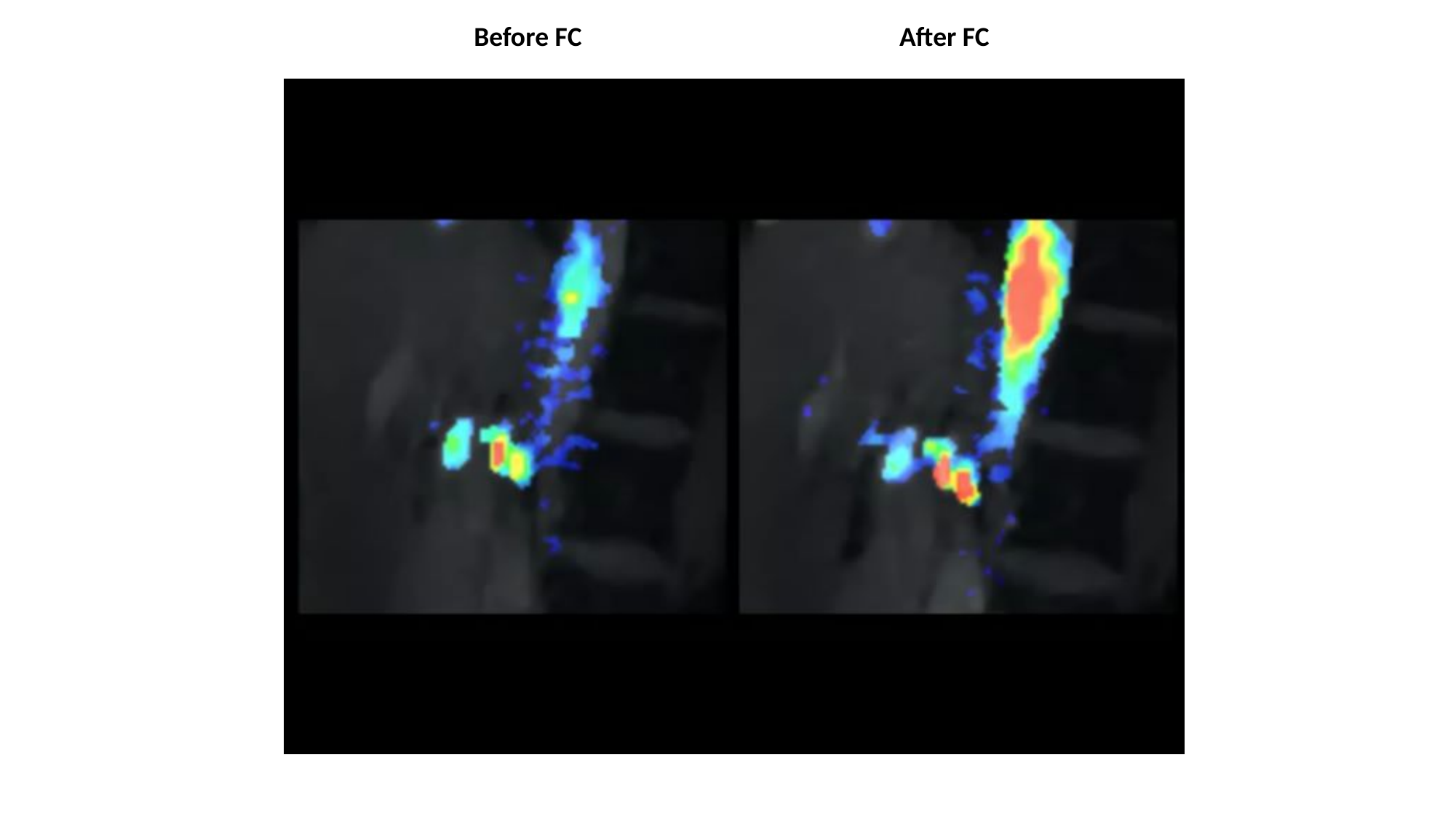

Before FC
After FC

Supplement: Supplementary file 3 [file Presentation1.PPTX]

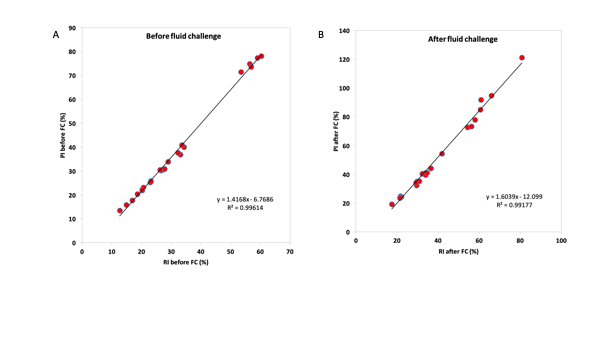

Supplement: Supplementary file 7 [file Image2.TIFF]
