# Supplementary material for: Portal Vein Pulsatility Index as a Potential Risk of Venous Congestion Assessed by Magnetic Resonance Imaging: A Prospective Study on Healthy Volunteers
Source: Front Physiol. 2022 Apr 29;13:811286. doi: 10.3389/fphys.2022.811286 (PMC9101294; doi:10.3389/fphys.2022.811286)
Supplement: Supplementary file 2 [file Table1.DOCX]

**Tableau 1 Demographics data**

Responder were defined by an increase in stroke volume of 10% after a fluid challenge of 500 ml. **BMI:** body mass index.

| **Variables** | **Non-responders**  **(n=10)** | **Responders**  **(n=14)** | **P value** |
| --- | --- | --- | --- |
| Age; *years* | 27 [25-28] | 28 [26-28] | 0.259 |
| BMI; *kg m^-2^* | 23.6 [22.1-24.3] | 23.4 [21.5-24.6] | 0.625 |
| body surface area; *m^2^* | 2.21 [2.05-2.24] | 1.99 [1.91-2.08] | 0.052 |
