# Supplementary material for: Portal Vein Pulsatility Index as a Potential Risk of Venous Congestion Assessed by Magnetic Resonance Imaging: A Prospective Study on Healthy Volunteers
Source: Front Physiol. 2022 Apr 29;13:811286. doi: 10.3389/fphys.2022.811286 (PMC9101294; doi:10.3389/fphys.2022.811286)
Supplement: Supplementary file 4 [file Table2.DOCX]

**Table 2 Clinical data, left ventricular (LV) and right ventricular (RV) echocardiographic parameters in non-responders and responders after 500 ml of fluid challenge (FC).**

**SAP:** systolic arterial pressure; **DAP:** diastolic arterial pressure; **MAP:** mean arterial pressure; **HR:** heart rate; **IVC:** inferior vena cava; **TAPSE:** tricuspid annular plane systolic excursion; **VTI:** velocity–time integral.

| **Variables** | **Non-responders**  **(n=10)** | **Responders**  **(n=14)** | **P value** |
| --- | --- | --- | --- |
| **Clinical data** | | | |
| SAP, *mmHg*  *Baseline*  *After FC* | 128 [125-137]  121 [119-126] | 118 [115-127]  117 [109-126] | 0.192  0.259 |
| DAP, *mmHg*  *Baseline*  *After FC* | 71 [62-84]  64 [60-75] | 64 [62-68]  66 [61-79] | 0.259  0.508 |
| MAP, *mmHg*  *Baseline*  *After FC* | 83 [80-87]  77 [72-85] | 76 [73-81]  77 [72-85] | **0.028**  0.886 |
| HR, *bpm*  *Baseline*  *After FC* | 67 [65-75]  62 [60-67] | 63 [60-73]  63 [56-68] | 0.508  0.796 |
| **LV echocardiography** | | | |
| VTI aortic, *cm s^-1^*  *Baseline*  *After FC* | 23.5 [22.0-24.0]  21.0 [18.0-23.0] | 19.0 [17.0-20.0]  24.5 [20.0-25.0] | **0.001**  0.108 |
| Stroke volume, *ml*  *Baseline*  *After FC* | 76 [71-91]  70 [62-97] | 65 [53-79]  78 [62-97] | **0.048**  0.508 |
| E wave, *cm s^-1^*  *Baseline*  *After FC* | 96 [80-101]  74 [62-93] | 76 [73-84]  88 [80-104] | **0.019**  0.508 |
| E/A ratio  *Baseline*  *After FC* | 1.9 [1.7-2.6]  2.2 [1.4-2.4] | 1.8 [1.5-1.9]  2.1 [1.7-2.6] | 0.312  0.977 |
| E deceleration time, *ms*  *Before*  *After FC* | 197 [171-234]  192 [157-283] | 236 [175-333]  234 [198-274] | 0.192  0.508 |
| Lateral E/e’  *Baseline*  *After FC* | 5.5 [4.0-6.0]  4.0 [4.0-4.0] | 4.0 [4.0-4.0]  5.0 [4.0-5.0] | 0.186  0.056 |
| **RV echocardiography** | | | |
| RV fractional area change, *%*  *Baseline*  *After FC* | 39 [35-43]  42 [35-45] | 48 [40-52]  43 [36-47] | 0.212  0.539 |
| TAPSE, *mm*  *Baseline*  *After FC* | 24 [21-29]  26 [23-29] | 22 [20-27]  25 [23-27] | **0.043**  0.508 |
| S wave, *cm s^-1^*  *Baseline*  *After FC* | 15 [14-17]  14 [13-16] | 14 [11-14]  14 [13-16] | 0.046  0.927 |
| IVC min diameter, *mm*  *Baseline*  *After FC* | 12 [10-14]  12 [11-14] | 11 [10-14]  16 [11-16] ^a^ | 0.841  0.212 |
| IVC max diameter, *mm*  *Baseline*  *After FC* | 20 [17-21]  21 [17-23] | 18 [16-22]  19 [17-22] | 0.585  0.472 |
| IVC collapsibility, *%*  *Baseline*  *After FC* | 38 [18-48]  36 [29-45] | 32 [23-50]  21 [11-36] | 0.709  0.074 |
| Sus hepatic S/D ratio  *Baseline*  *After FC* | 1.33 [1.07-1.55]  1.35 [1.26-1.67] | 1.16 [0.96-1.39]  1.25 [1.05-1.58] | 0.600  0.285 |
