# Supplementary material for: Portal Vein Pulsatility Index as a Potential Risk of Venous Congestion Assessed by Magnetic Resonance Imaging: A Prospective Study on Healthy Volunteers
Source: Front Physiol. 2022 Apr 29;13:811286. doi: 10.3389/fphys.2022.811286 (PMC9101294; doi:10.3389/fphys.2022.811286)
Supplement: Supplementary file 5 [file Table3.DOCX]

**Table 3 Comparisons of MRI portal haemodynamic between non-responders and responders after 500 ml of fluid challenge.**

| **Variables** | **Non-responders**  **(n=10)** | **Responders**  **(n=14)** | ***P* value** |
| --- | --- | --- | --- |
| **Portal flow, *ml min^-1^***  Baseline  After FC | 1042 [986-1287]  1034 [961-1273] | 881 [762-1001]  1010 [778-1106] ^a^ | **0.022**  0.371 |
| **Portal velocity, *cm s^-1^***  Baseline  After FC | 24.0 [20.8-29.9]  26.4 [22.1-30.1] | 20.8 [18.7-26.6]  21.4 [19.6-26.9] | 0.122  0.212 |
| **Pulsatility index, *%***  Baseline  After FC | 32 [22-40]  48 [25-85] ^a^ | 31 [25-41]  35 [25-42] | 0.931  0.312 |
| **Portal vein cross-sectional area, *cm^2^***  Baseline  After FC | 1.7 [1.6-1.9]  1.8 [1.5-2.1] | 1.5 [1.4-1.7]  1.6 [1.5-1.7] | 0.064  0.437 |

Data are expressed as median [interquartile range]. **SV:** stroke volume. **Pulsatility index** was calculated as 100*(maximum portal velocity – minimum portal velocity)/maximum portal velocity. **a:** *P* value<0.05 for baseline/post-FC comparisons using the Wilcoxon rank sum test.
