# Supplementary material for: Portal Vein Pulsatility Index as a Potential Risk of Venous Congestion Assessed by Magnetic Resonance Imaging: A Prospective Study on Healthy Volunteers
Source: Front Physiol. 2022 Apr 29;13:811286. doi: 10.3389/fphys.2022.811286 (PMC9101294; doi:10.3389/fphys.2022.811286)
Supplement: Supplementary file 6 [file Table4.DOCX]

**Extra supplemental table 1. Pulsatility index at 15 and 20% of stroke volume variation (SVV) after fluid challenge (FC).**

| **Responder with SVV>15%** | | | |
| --- | --- | --- | --- |
| **PI, *%*** | Non-responder  (n=14) | Responder  (n=10) | *P*- value |
| *Before FC* | 32 [22-40] | 30 [25-41] | 0.886 |
| *After FC* | 41 [25-78] * | 34 [25-42] | 0.472 |
| **Responder with SVV >20%** | | | |
| **PI, *%*** | Non-responder  (n=17) | Responder  (n=7) | *P*- value |
| *Before FC* | 31 [22-38] | 31 [26-43] | 0.318 |
| *After FC* | 42 [25-72] * | 35 [28-58] | 0.664 |

Before FC, PI was similar between responder at 15 % of SVV and 20 of SVV%. After FC, PI significantly increased in non-responders at 15% of SVV and 20 of SVV%. However, PI did not differ between groups after FC.
